# A Universal Deep Learning Model for Predicting Detection Performance and Single-Event Effects of SPAD Devices

**DOI:** 10.3390/mi17040452

**Published:** 2026-04-07

**Authors:** Yilei Chen, Jin Huang, Yuxiang Zeng, Yi Jiang, Shulong Wang, Shupeng Chen, Hongxia Liu

**Affiliations:** School of Microelectronics, Xidian University, Xi’an 710071, China; ylchen10969@163.com (Y.C.); 23111213500@stu.xidian.edu.cn (Y.Z.); jiang_yi1113@163.com (Y.J.); spchen@xidian.edu.cn (S.C.); hxliu@mail.xidian.edu.cn (H.L.)

**Keywords:** single-event effect (SEE), deep learning (DL), single-photon avalanche diode (SPAD), technology computer-aided design (TCAD)

## Abstract

Single-event effects (SEEs) present a significant challenge to the radiation reliability of integrated circuits. Conventional SEE analysis methods for single-photon avalanche diode (SPAD) devices primarily rely on Sentaurus Technology Computer-Aided Design (TCAD) numerical simulation, which is computationally intensive and time-consuming. In this study, we propose a generalized deep learning (DL) model, using a silicon-based SPAD device with a double-junction double-buried-layer (DJDB) structure fabricated in 180 nm CMOS process as the research subject. By incorporating key parameters that influence SEEs as model inputs, the proposed approach enables rapid prediction of critical parameter metrics, including transient current peaks and dark count rates. Experimental results show that the DL model achieves a prediction accuracy of 97.32% for transient current peaks and 99.87% for dark count rates, demonstrating extremely high prediction precision. To further validate the generalization capability of the proposed network, the model is applied to predict the detection performance of the DJDB-SPAD device. The prediction accuracies for four key performance parameters all exceed 97.5%, further confirming the accuracy and robustness of the developed model. Meanwhile, compared with the conventional Sentaurus TCAD simulation method, the proposed method achieves a 336-fold improvement in computational efficiency. Overall, this method realizes the dual advantages of high precision and high efficiency, which provides an efficient and accurate technical solution for the rapid characteristic analysis and reliability evaluation of SPAD devices under single-event effects (SEEs).

## 1. Introduction

Single-event effects (SEEs) refer to functional anomalies in microelectronic devices induced by high-energy particle irradiation. When cosmic rays, alpha particles, or protons interact with semiconductor materials, electron–hole pairs are generated through ionization processes. If these charge carriers are produced near sensitive circuit nodes, they can induce transient current spikes under the influence of the internal electric field. When the collected charge exceeds the critical charge threshold (Qcrit) [1,2,3], bit flips or other functional failures may occur. This stochastic reliability issue remains a central concern in enhancing the robustness of integrated circuits.

Current SEE research on photodetectors primarily concentrates on radiation-induced dark current degradation. In 2017, Kumar et al. [4] irradiated silicon photodetectors (N+/P/P+) and observed significant electrical degradation at a total ionizing dose of 60 Mrad, accompanied by an increase in reverse dark current. In 2019, Sandia National Laboratories reported elevated internal dark current in Ge-Si photodiodes under reverse bias following ^60^Co γ-ray irradiation. These experimental investigations examined various high-energy particles (e.g., heavy ions and protons) and systematically analyzed SEE mechanisms and influencing factors. The results demonstrated that particle energy, charge state, and incidence angle significantly affect the severity of SEE.

Physical irradiation experiments tend to cause irreversible damage to devices and suffer from high cost and long cycles, making it difficult to support large-scale parametric iterative research. Sentaurus Technology Computer-Aided Design (TCAD) is the mainstream tool for semiconductor device simulation. Although numerical simulations based on Sentaurus TCAD can accurately reveal the internal physical mechanisms of devices, they suffer from large computational burden and low efficiency, meaning they can hardly meet the practical demands for rapid reliability evaluation and design optimization of devices.

In view of the limitations of conventional experimental and numerical simulation methods in terms of efficiency, cost, and multi-dimensional analysis capability, an increasing number of researchers have applied intelligent algorithms to the study of irradiation effects. Artificial intelligence (AI), particularly neural networks that emulate human cognitive processes for data interpretation and pattern recognition, has achieved substantial progress in electronic design automation [5,6,7,8,9,10]. These computational techniques offer advantages in modeling flexibility and accelerated simulation throughput, demonstrating broad applicability across disciplines such as quantum systems, photonic materials, nanoscale device architectures, and semiconductor technologies.

In semiconductor device research, deep learning methods have been widely adopted across various applications [11,12,13,14,15]. Deep learning is an important branch of machine learning, whose core idea is to automatically learn features and underlying rules from massive data by constructing multi-layer neural network structures. Chen et al. [16] developed a deep neural network model to predict the breakdown characteristics of silicon-on-insulator (SOI) lateral power devices, achieving a mean prediction error below 4%. Akbar et al. [17] systematically investigated the impact of multiple variability sources on semiconductor nanodevice performance using deep learning techniques. Furthermore, Hirtz et al. [18] employed convolutional neural networks for multi-current–voltage (I-V) characteristic analysis, demonstrating the capability to efficiently emulate semiconductor process variations while significantly reducing simulation time.

This paper proposes a deep learning-based method for predicting single-event effects (SEEs). The input features of the network include the linear energy transfer (LET) of the incident particle, the particle incidence position (x), the incident angle (θ), and the excess bias voltage (V_ex_). A deep neural network (DNN), which has been widely adopted in semiconductor device research, is employed as the predictive model. The network outputs two key parameters: the transient current peak (I_0_) and the dark count rate (DCR).

The predictive performance of the model is evaluated using multiple metrics, including accuracy, mean square error (MSE), and the coefficient of determination (R^2^). The proposed approach demonstrates high computational efficiency and strong predictive accuracy, enabling rapid SEE assessment without requiring detailed device physics modeling.

## 2. Experimental

### 2.1. Device Structure

**Figure 1:**
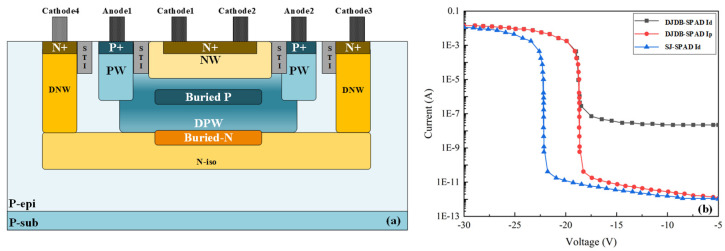
Structure of the proposed DJDB-SPAD device and its electrical characteristics. (**a**) Schematic cross-sectional view of the DJDB-SPAD device structure. (**b**) Simulated current–voltage (I-V) characteristics of the DJDB-SPAD and conventional single-junction SPAD (SJ-SPAD), obtained using Sentaurus TCAD.

The novel double-junction double-buried-layer SPAD (DJDB-SPAD) structure investigated in this study is illustrated in Figure 1a. Unlike conventional SPAD architectures, the proposed device adopts a composite N+/N-well/N-isolation layer configuration formed on a silicon substrate. The N-isolation layer provides electrical isolation between adjacent devices to suppress optical crosstalk. An N-buried layer is subsequently introduced above the isolation layer through ion implantation. A P-buried layer is then formed within the deep P-well via ion implantation followed by thermal annealing to tailor the electric field distribution.

Between the deep P-well and the deep N-well, P+/P-well structures are fabricated through diffusion and ion implantation. The P+ region functions as the anode, while the adjacent P-well regions reduce the on-resistance. A lightly doped epitaxial layer located between the P-well and deep N-well acts as a virtual guard ring to alleviate edge electric field crowding. In addition, shallow trench isolation (STI) structures are implemented on both sides of the P-well to suppress premature edge breakdown. The detailed structural parameters are summarized in Table 1.

Figure 1b presents a comparison of the simulated current–voltage (I-V) characteristics of a conventional single-junction SPAD (PW/DNW) and the proposed double-junction double-buried-layer SPAD (NW/PBL/DPW/NBL), obtained using Sentaurus TCAD 2017. Dark current simulations indicate that the reference SPAD exhibits an avalanche breakdown voltage of 22.1 V, whereas the proposed structure shows a reduced breakdown voltage of 18.8 V. This reduction implies a higher achievable excess bias voltage under identical operating conditions, thereby enhancing photon detection efficiency (PDE), given the positive correlation between overbias and PDE.

### 2.2. Dataset

Sentaurus TCAD was employed to generate the simulation dataset. The selected network input parameters include the linear energy transfer (LET) of the incident particle, the particle incident position (x), the incident angle (θ), and the excess bias voltage (V_ex_). For SEE prediction, the output characterization metrics are defined as the transient current peak (I_0_) and dark count rate (DCR).

After specifying the input and output parameters, Sentaurus TCAD simulations were conducted for dataset generation. The fundamental physical models incorporated in the simulations include: (1) Poisson’s equation, carrier continuity equations, and carrier transport equations; (2) Fermi–Dirac statistics; (3) Shockley–Read–Hall (SRH) and Auger recombination models; (4) the heavy-ion charge density model; and (5) a Gaussian-distributed heavy-ion irradiation model.

**Figure 2:**
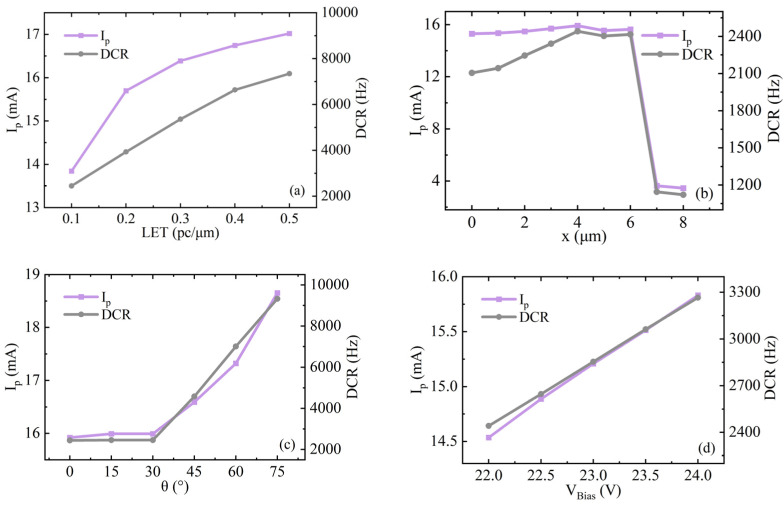
Influence of (**a**) linear energy transfer (LET), (**b**) particle incidence position (x), (**c**) particle incidence angle (θ), and (**d**) excess bias voltage (V_ex_) on the transient current peak (I_0_) and dark count rate (DCR).

Figure 2 illustrates the influence of the four input parameters on the transient current peak under specified conditions (LET = 0.1 pc/μm, default incident position = 0 μm, incident angle = 0°, and excess bias voltage = 4 V). As observed, all four parameters significantly affect both I_0_ and DCR. Notably, LET and V_ex_ exhibit approximately linear correlations with I_0_ and DCR, respectively.

When particles are incident at x = 4 μm (corresponding to the N-well edge), the cathode transient pulse reaches its maximum peak value. This position coincides with the boundary of the avalanche multiplication region. Particle incidence at this location enhances the local electric field at the edge of the avalanche region, thereby facilitating the drift and collection of generated electron–hole pairs.

Both the transient current peak and pulse width attain their maximum values when the particle incidence angle θ is 75°. This behavior arises because the transient current is strongly dependent on the ion track length within the depletion region. For 0 ≤ θ ≤ 30°, the relatively small incidence angles result in minimal variation in ion penetration depth across the depletion region. In contrast, when θ ≥ 45°, larger incidence angles produce extended ion tracks within the depletion region, generating a greater number of electron–hole pairs that drift under the strong electric field.

Based on these parameter configurations, single-event effects (SEEs) in the SPAD device are systematically simulated to obtain the dataset required for subsequent network training.

Due to the absence of available existing datasets, this study utilized Sentaurus TCAD simulation software 2017 to generate a total of 2683 simulation cases for the training and validation of the neural network. Given the complexity of single-event effect simulations, the entire simulation process took more than 80 h.

The configuration of the parameter space is summarized in Table 2. Considering the structural symmetry of the SPAD device, sampling was performed on half of the device structure, with the center of symmetry defined as the origin (x = 0). Here, θ denotes the angle between the particle trajectory and the surface normal.

The dataset was randomly divided into three subsets: 80% was used for training, 10% for validation, and the remaining 10% for testing. Prior to network training, the input features were normalized to the [0, 1] interval using min-max scaling [19,20]. The normalization process is mathematically expressed in Equation (1).

(1) $$x^{*}=\frac{x-\min\left(x\right)}{\max\left(x\right)-\min\left(x\right)}$$

Here, x denotes the input feature, while min(x) and max(x) represent the lower and upper bounds of the observed data distribution, respectively. This normalization strategy ensures that all features share a consistent numerical scale while preserving their intrinsic distribution characteristics. Data normalization is critical because neural networks inherently learn underlying data distributions. Discrepancies between training and test distributions can significantly degrade model generalization performance [21]. Moreover, inconsistent feature distributions across mini-batches require continuous parameter adaptation during training, thereby slowing convergence [22,23,24,25].

## 3. Results and Discussion

### 3.1. Prediction of Transient Current Peak and DCR

**Figure 3:**
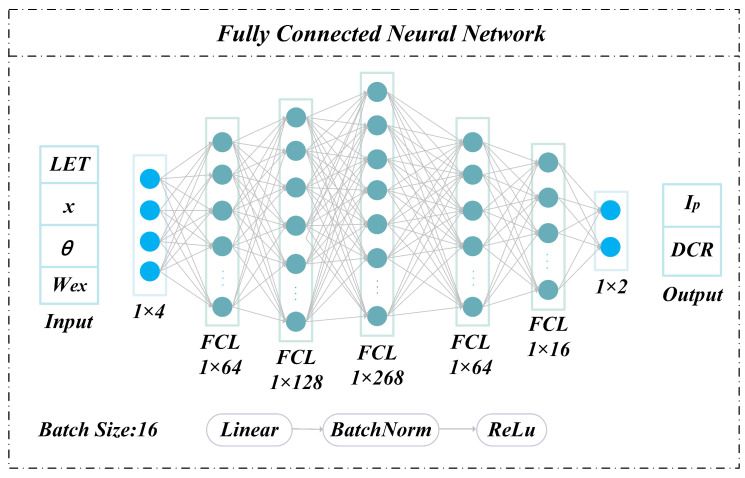
Network structure for predicting transient current peak (I_0_) and dark count rate.

The network architecture for predicting transient current peaks and dark count rates is shown in Figure 3.

The deep neural network (DNN) consists of five fully connected layers for training and inference. Each layer comprises a linear transformation layer, a batch normalization (BN) layer, and a Rectified Linear Unit (ReLU) activation layer. The linear layer performs affine transformations of the inputs. The BN layer mitigates internal covariate shift, stabilizes inter-layer feature distributions, accelerates convergence, and introduces a regularization effect similar to noise injection, thereby reducing overfitting [26,27,28].

The ReLU is the most commonly used activation function in deep learning, with the mathematical form [29]:

(2) f(x)=max(0,x)

It sets the input values less than 0 directly to 0 and keeps those greater than or equal to 0 unchanged. It introduces nonlinearity, improves computational efficiency, and alleviates gradient vanishing and explosion issues [19,30]. Early stopping (patience = 30 epochs) is applied by monitoring validation loss to further prevent overfitting.

The transient current peak I_0_ is defined in Equation (3), where q denotes the elementary charge, $\mu_{n}$ is the electron mobility, N_a_ is the channel doping concentration, N is the line density of generated electron–hole pairs, ε is the dielectric constant, and x_p_ is the depletion width of the PN junction.

(3) $$I_{0}=\frac{q^{2}\mu_{n}N_{a}N}{\varepsilon }x_{p}$$

The dark count rate (DCR) is given in Equation (4), where W_1_ and W_2_ denote the upper and lower boundaries of the avalanche depletion region, P_tr_(x) represents the carrier-triggered avalanche probability at depth x, and S is the active area.

(4) $$DCR=S\int_{W_{1}}^{W_{1}+W_{2}}P_{tr}\left(x\right)\cdot \left(G_{SRH,TAT}+G_{BBT}\right)dx$$

**Figure 4:**
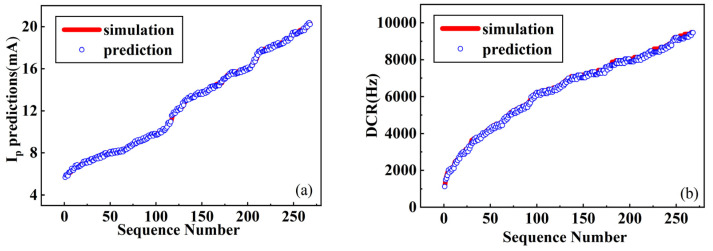
Results of predicting the transient current peak and dark count rates. (**a**) Comparison between simulated and predicted I_0_; (**b**) comparison between simulated and predicted DCR.

Using the network model shown in Figure 3, training and evaluation were completed based on 2683 data samples. The trained network model was tested on 268 samples from the test set, with a total computation time of 85.66 s, representing a 336-fold efficiency improvement over Sentaurus TCAD simulation. The prediction performance is illustrated in Figure 4. Figure 4a and Figure 4b compare predicted and TCAD-simulated values of I_0_ and DCR across 268 test samples, respectively. The red line denotes simulation results, while blue markers represent network predictions; the vertical distance between them indicates prediction error. When sorted in ascending order of simulated values, the predictions closely track the baseline with only minor fluctuations, demonstrating strong fitting capability.

**Figure 5:**
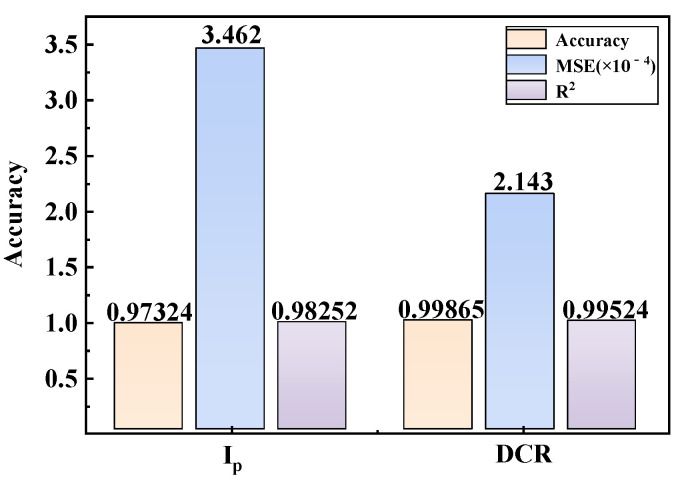
Prediction accuracy and error analysis of single-event characterization parameters for SPAD.

Prediction performance is quantified using accuracy, mean squared error (MSE), and linear regression coefficients. As shown in Figure 5, the model achieves accuracies of 97.32% and 99.87% for I_0_ and DCR, respectively, with MSE values of 3.462 × 10^−4^ and 2.143 × 10^−4^, and regression coefficients of 0.983 and 0.995. These results confirm the high-precision predictive capability of the proposed network for SEE characterization parameters.

### 3.2. Prediction of Detection Performance for Double-Junction Double-Buried-Layer SPAD

To further evaluate generalization capability, the network was applied to predict detection performance metrics of double-junction double-buried-layer SPAD devices. The input features include P-buried-layer depth (T_p_), P-buried-layer doping concentration (N_bp_), deep P-well doping (N_dp_), N-buried-layer radius (R_bn_) and N-buried-layer doping concentration (N_bn_). The output parameters include breakdown voltage (V_BR_), dark count rate (DCR), avalanche probability (P), and photon detection efficiency (PDE) at 850 nm. The corresponding input configurations are listed in Table 3.

**Figure 6:**
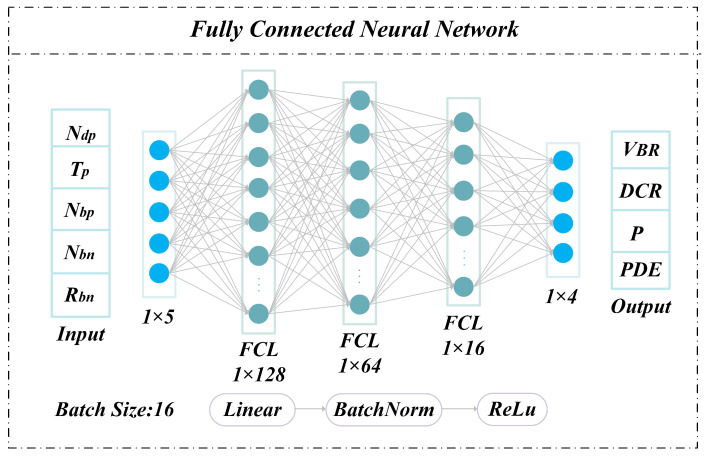
Structure of the novel neural network model for predicting the detection performance of SPAD.

The network structure was adjusted accordingly in response to changes in input–output dimensionality, while the same architectural principles as those in Figure 3 were retained. The universality of the proposed fully connected neural network framework is mainly reflected in three aspects. First, all layers adopt fully connected layers, which achieve efficient feature extraction and mapping while comprehensively balancing computational resource consumption and model expressive power, and avoid the parameter explosion caused by convolutional layers [31,32,33]. Secondly, the modular structure of “Linear transformation + Batch Normalization (BatchNorm) + ReLU activation” is consistently employed across all tasks, ensuring the consistency and reusability of the architecture. Finally, the network can be adapted to the input–output dimensions of different tasks only by adjusting the number of neurons in each fully connected layer (FCL), without the need to reconstruct the core network logic. The modified network structure is shown in Figure 6. Apart from the neuron count and the number of fully connected layers being adapted to the feature dimensionality, the overall structure remains consistent with the single-event effect (SEE) prediction model.

**Figure 7:**
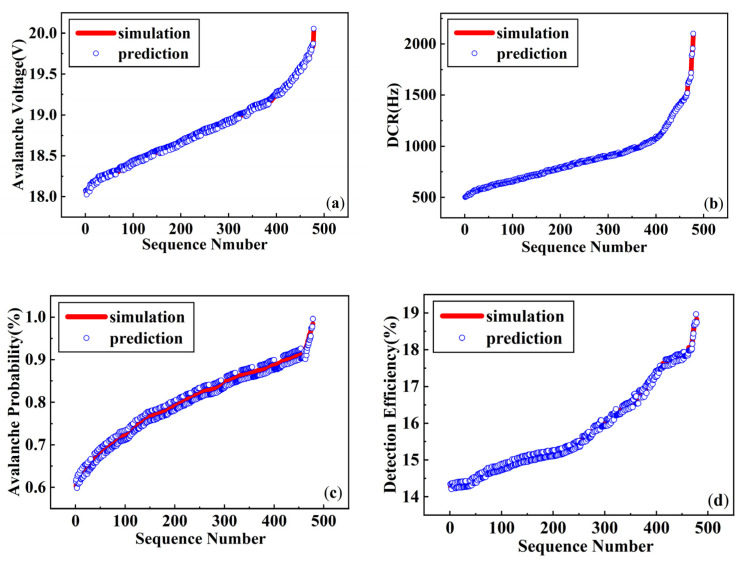
Prediction results of novel SPAD detection performance: (**a**) prediction results of avalanche breakdown voltage; (**b**) prediction results of dark count rate; (**c**) prediction results of avalanche probability; and (**d**) prediction results of detection efficiency in 850 nm.

The optimized network was adopted to predict 497 test samples. For analytical convenience, the simulated values in the test set were sorted in ascending order. Comparative diagrams depicting the simulated and predicted values of avalanche breakdown voltage, dark count rate, and detection efficiency at 850 nm were generated, as shown in Figure 7. It is evident that the predicted values fluctuate slightly around the simulated values, indicating that the network model exhibits excellent fitting performance for all four parameter sets.

**Figure 8:**
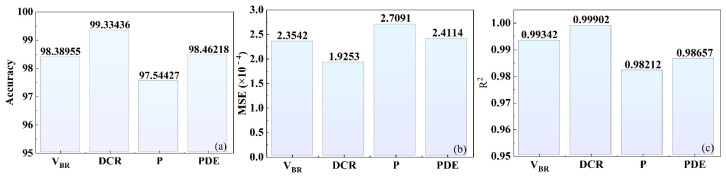
Prediction accuracy and error diagram of detection performance parameters: (**a**) accuracy; (**b**) mean squared error; and (**c**) linear regression factor.

To provide a more intuitive evaluation of the predictive performance of the proposed DNN for the double-junction double-buried-layer SPAD, Figure 8 shows the prediction accuracy, mean squared error (MSE), and linear fit coefficient for each parameter in the test set. The prediction accuracies of the avalanche breakdown voltage, dark count rate, avalanche probability, and photon detection efficiency at 850 nm are 98.39%, 99.33%, 97.54%, and 98.46%, respectively.

Overall, the model delivers superior predictive performance for the dark count rate, avalanche breakdown voltage, and photon detection efficiency at 850 nm, whereas the prediction performance for avalanche probability is slightly lower. This discrepancy is primarily attributable to differences in the dynamic ranges of the simulated values: the first three parameters exhibit considerably larger variations, with the dark count rate in particular having a range exceeding 55 Hz, while avalanche probability varies by only 0.6. Such a narrow dynamic range can reduce the apparent prediction accuracy because even minor fluctuations in the network outputs may result in relatively large normalized errors. Although logarithmic transformation improves the separability of the data to a certain extent, the overall dynamic range remains small. Nevertheless, the prediction for avalanche probability still achieves an accuracy of 97.54%, an MSE of 2.71, and a linear regression coefficient of 0.98—demonstrating that the proposed model offers sufficient accuracy for research focused on predicting the detection performance of SPAD devices.

## 4. Conclusions

This paper proposes a deep learning-based framework for predicting single-event effects (SEEs) in single-photon avalanche diode (SPAD) devices. First, we investigate the effects of linear energy transfer (LET), particle incidence position, incident angle, excess bias voltage on transient current, and dark count rate (DCR). The results demonstrate that both the transient current peak and DCR increase with elevated LET values, incidence angles, and excess bias voltage. For incident positions, the transient pulse peaks occur at the edges of the avalanche region, while maximum pulse widths emerge near P-wells. Accordingly, a neural network architecture is designed for predicting the transient current peak and DCR, with its inputs consisting of key SEE parameters: LET of incident particles, incident position (x), incident angle (θ), and excess bias voltage (Vex). The model achieves a prediction accuracy of 97.32% for the transient current peak and 99.87% for DCR, with the linear regression coefficients reaching 0.993 and 0.995, respectively. These metrics validate the high-precision predictive performance of the neural network for SPAD performance parameters.

To verify the general applicability of this network for SPAD device performance prediction, the model is further applied to predict the detection performance of double-junction double-buried-layer SPADs. The prediction accuracies for the four key performance parameters reach 98.39%, 99.33%, 97.54%, and 98.46%, respectively, which further confirms the accuracy and reliability of the proposed model.

This data-driven framework provides an efficient and transferable paradigm for SEE analysis and broader semiconductor device modeling applications.

## Figures and Tables

**Table 1:** DJDB-SPAD device parameters.

Parameters	Values
P-buried layer depth (T_p_)	1.6 μm
N-buried layer radius (R_bn_)	4 μm
P-buried layer doping concentration (N_bp_)	3.1 × 10^17^ cm^−3^
N-buried layer doping concentration (N_bn_)	4.8 × 10^17^ cm^−3^
P substrate doping concentration	1 × 10^19^ cm^−3^
P-epitaxial layer doping concentration	5 × 10^14^ cm^−3^
P-well doping concentration	1 × 10^17^ cm^−3^
N-well doping concentration	1 × 10^17^ cm^−3^
N-isolation layer doping concentration	5 × 10^16^ cm^−3^

**Table 2:** Input parameters of the deep neural network.

Input Parameters	Range/Step
Linear transmission energy (LET)	[0.05, 0.5]/0.05 (pc/μm)
The incident position of the particle (x)	[0, 8]/1 (μm)
The incident angle of the particle (θ)	[0, 75]/15 (°)
The excess bias voltage (Vex)	[3, 5]/0.5 (V)

**Table 3:** Input parameters of deep neural network for predicting the performance of SPAD.

Input Parameters	Values
The input vector incorporated P-buried layer depth (T*p*)	1.1, 1.2, 1.3, 1.4, 1.5, 1.6, 1.7, 1.8 (μm)
P-buried layer doping concentration (N*bp*)	2.0, 2.5, 3.0, 3.5, 4.0 (×10^17^ cm^−3^)
Deep P-well doping (N*dp*)	6, 8, 10, 13, 15 (×10^16^ cm^−3^)
The N-buried layer radius (R*bn*)	2, 3, 4, 5, 6 (μm)
N-buried layer doping concentration (N*bn*)	2.5, 5.0, 7.5, 10.0, 12.5 (×10^17^ cm^−3^)

## Data Availability

The original contributions presented in this study are included in the article. Further inquiries can be directed to the corresponding author.
